# Correlation of electrocardiographic conduction abnormalities with myocardial fibrosis and scar in late enhancement

**DOI:** 10.1186/1532-429X-17-S1-P189

**Published:** 2015-02-03

**Authors:** Olaf C Grebe, Christa Voemel, Bernward K Kurtz, Ernst G Vester

**Affiliations:** Cardiology, EVK - Evangelist Hospital Duesseldorf, Duesseldorf, Germany; Radiology, EVK - Evangelist Hospital Duesseldorf, Duesseldorf, Germany

## Background

Late Gadolinium enhancement represents a widely employed method for detecting myocardial scarring e.g. after myocardial infarction or for myocardial Inflammation. A frequent finding is slight basal septal enhancement as well as intramyocardial enhancement, mostly referred as myocardial fibrosis. Less is known about the impact on myocardial conduction and a possible correlation to ECG findings such as atrioventricular node conduction delay and bundle branch blockations.

## Methods

For 81 consecutive patients (mean age 63,5yrs, range 20-84yrs, 17 female) referred for magnetic resonance cardiac (MRI) including a late gadolinium enhancement study scans were anonymized and correlated to an ECG performed within one week before or after the MRI. A 1.5T Siemens Sonata with TIM upgrade was used. Scanning protocol comprised short- and long axis cine mode and a perfusion study with Gd-DOTA and/or T1 and T2 TSE as well as 2d and 3d IR short axis sequences for late enhancement. The latter was analysed visually for the study by two experienced operators and subdived as (1) normal, (2) intramyocardial fibrosis, (3) subendocardial or transmural ischemic scar and (4) bright acute inflammation or postmyocardial subepicardial enhancement pattern. The ECGs had been re-evaluated by an experienced cardiologist.

## Results

The baseline data between the 4 groups was similar (data given as mean values and range):

group 1 (normal): 30 patients (37%), LVEF 62% (17-79%), EDVI 73ml (40-222ml), ventricular septum thickness (IVS) 11mm, age 57yrs;

group 2 (fibrosis): 30 patients (37%), LVEF 58% (20-76%), EDVI 72ml (45-142ml), IVS 13mm, age 68yrs;

group 3 (ischemic scar): 15 patients (19%), LVEF 51% (30-76%), EDVI 82ml (36-152ml), IVS 11mm, age 69yrs;

group 4 (myocarditis): 6 patients (7%), LVEF 55% (42-71%), EDVI 82ml (57-122ml), IVS 10mm, age 44yrs.

Atrial fibrillation was present in 10%, 20%, 27%, and 0% respectively.

ECG findings are presented in Table 1. Group 2-3 differed significantly from each other (p<0.016) as well as the fibrosis vs. the normal group p<0.01).

**Table 1:** ECG findings

	normal ECG	LBBB	RBBB	HB	IVCD	AVB
normal	83%	0%	17%	10%	0%	7%
fibrosis	47%	10%	10%	13%	27%	20%
ischemic scar	47%	20%	7%	0%	27%	20%
myocarditis	33%	0%	50%	17%	0%	0%

LBBB/RBBB: left/right bundle branch blockation; HB: left hemi blockation; IVCD intraventricular conduction delay, AVB: atrioventricular node block (all grade I)

## Conclusions

Abnormal ECG findings such as AV conduction delay and bundle branch blockation are significantly correlated to myocardial scarring or fibrosis in late enhancment studies.

## Funding

None.

